# Genomic prediction based on a joint reference population for the Xinjiang Brown cattle

**DOI:** 10.3389/fgene.2024.1394636

**Published:** 2024-04-26

**Authors:** Menghua Zhang, Lei Xu, Haibo Lu, Hanpeng Luo, Jinghang Zhou, Dan Wang, Xiaoxue Zhang, Xixia Huang, Yachun Wang

**Affiliations:** ^1^ College of Animal Science, Xinjiang Agricultural University, Urumqi, China; ^2^ Laboratory of Animal Genetics, Breeding and Reproduction, Ministry of Agriculture of China, National Engineering Laboratory of Animal Breeding, College of Animal Science and Technology, China Agricultural University, Beijing, China; ^3^ Shijiazhuang Molbreeding Biotechnology Co., Ltd., Shijiazhuang, China

**Keywords:** Xinjiang Brown cattle, multi-breed, genomic prediction, bayes, single-step GBLUP

## Abstract

**Introduction:** Xinjiang Brown cattle constitute the largest breed of cattle in Xinjiang. Therefore, it is crucial to establish a genomic evaluation system, especially for those with low levels of breed improvement.

**Methods:** This study aimed to establish a cross breed joint reference population by analyzing the genetic structure of 485 Xinjiang Brown cattle and 2,633 Chinese Holstein cattle (Illumina GeneSeek GGP bovine 150 K chip). The Bayes method single-step genome-wide best linear unbiased prediction was used to conduct a genomic evaluation of the joint reference population for the milk traits of Xinjiang Brown cattle. The reference population of Chinese Holstein cattle was randomly divided into groups to construct the joint reference population. By comparing the prediction accuracy, estimation bias, and inflation coefficient of the validation population, the optimal number of joint reference populations was determined.

**Results and Discussion:** The results indicated a distinct genetic structure difference between the two breeds of adult cows, and both breeds should be considered when constructing multi-breed joint reference and validation populations. The reliability range of genome prediction of milk traits in the joint reference population was 0.142–0.465. Initially, it was determined that the inclusion of 600 and 900 Chinese Holstein cattle in the joint reference population positively impacted the genomic prediction of Xinjiang Brown cattle to certain extent. It was feasible to incorporate the Chinese Holstein into Xinjiang Brown cattle population to form a joint reference population for multi-breed genomic evaluation. However, for different Xinjiang Brown cattle populations, a fixed number of Chinese Holstein cattle cannot be directly added during multi-breed genomic selection. Pre-evaluation analysis based on the genetic structure, kinship, and other factors of the current population is required to ensure the authenticity and reliability of genomic predictions and improve estimation accuracy.

## 1 Introduction

Xinjiang Brown cattle is a major breed supporting the development of the cattle industry in Xinjiang, it was the first breed of cattle used for milk and meat purposes after the founding of the People’s Republic of China. In 2023, the number of Xinjiang Brown cattle in stock reached 1.16 million; however, the level of breed improvement was low, with a performance measurement population of <10,000. Therefore, it is important to establish an efficient genomic evaluation system for Xinjiang Brown cattle to improve their genetic level. The application of genome selection technology has significantly enhanced the efficiency of genomic evaluation (Hayes et al., 2016). Because of the implementation of the genome selection for Chinese Holstein cattle in 2008, early and accurate selection of calves and young cattle has been achieved (George et al., 2017), leading to higher accuracy in genomic evaluation and more precise assessment of individual breeding value (Weigel et al., 2010; Dassonneville et al., 2011). In addition, due to early selection and higher accuracy, the rate of genetic progress has doubled (Weller et al., 2017), improving breeding profitability and significantly reducing breeding costs. Although genome selection has been successfully applied to Chinese Holstein cattle population, the low level of production performance measurement and small population size of Xinjiang Brown cattle have hindered the application of genome selection technology. To improve the reliability of genomic predictions, especially for smaller populations, many feasible methods have been proposed, including increasing marker density, constructing linkage disquilibrium (LD) with more markers and causal mutations, and simulation data analysis (de Roos et al., 2009). Simulation and real data analyses (BrØndum et al., 2015) have shown that genomic prediction can play an important role in different populations.

For genome selection, it is necessary to have a reference population with sufficient size and an appropriate genetic structure that simultaneously incorporates genomic and phenotypic information to accurately predict genome estimated breeding values (GEBVs) (Metta et al., 2004; Boichard et al., 2016). Genome selection has recently been widely used in dairy cattle breeding programs. However, its application is limited to populations with a small number of breeds. Establishing a sufficiently large reference population is the most limiting factor for the accurate estimation of SNP effects (Boichard et al., 2016). When conducting genome selection for small populations, the most direct approach to enhancing its reliability is to expand the reference population. Many countries have found effective solutions through international cooperation, leading to joint genomic evaluations (Lund et al., 2011). By connecting France, Germany, Austria, Italy, Slovenia, Switzerland, and the United States of America to the InterGenomics consortium operated by the Interbull Center (Zumbach et al., 2010; Jorjani et al., 2012), genome-wide joint evaluations have been conducted for Brown Swiss bulls and Simmental cattle in Germany and Austria (Edel et al., 2011). Research has shown that by combining different populations of the same breed or related breeds in the reference population, more effective information can be obtained for estimating marker effects. Therefore, more accurate breeding predictions can be obtained from genomic predictions. Accuracy is improved when three related dairy cattle populations, Danish Red, Swedish Red, and Finnish Ayrshire, are combined into a single reference population (Zhou et al., 2014). When four European Holstein populations were combined into a reference population, the reliability increased by 10% (Lund et al., 2011). By combining six Brown Swiss populations, the reliability increased from 6% to 45% (Jorjani et al., 2012). However, multi-breed genomic evaluation of Xinjiang Brown cattle has not yet been conducted, limiting the optimized utilization of genomic selection technology in their genomic evaluation.

Based on the research foundation for domestic and international multi-breed joint genomic evaluation (Pryce et al., 2011; Lund et al., 2014; Steyn et al., 2019; Xu et al., 2019; Palombo et al., 2021), we proposed to integrate Xinjiang Brown cattle and Chinese Holstein cattle to construct a joint reference population for genome selection. In order to expand the Xinjiang brown cattle genome selection reference group, so as to apply multi-breed genome selection in Xinjiang brown cattle population to improve the prediction reliability. This study aimed to analyze the genetic structures of Xinjiang Brown and Chinese Holstein cattle to establish a multi-breed joint reference population. Using a dual-trait single-step genome-wide best linear unbiased prediction (ssGBLUP) approach, we established a genomic evaluation system for the primary lactation traits of Xinjiang Brown cattle, leveraging the joint reference population of Xinjiang Brown and Chinese Holstein cattle. This improves the accuracy of genomic selection for Xinjiang Brown cattle, creating a core breeding herd of genetically superior dairy Xinjiang Brown cows. Consequently, the genetic improvement of Xinjiang Brown cattle population will be expedited, leading to enhanced genetic levels across the breed.

## 2 Materials and methods

### 2.1 Sample collection and DNA extraction

A total of 1,729 blood samples were collected from the tail vein of Xinjiang Brown cattle and added to 10 mL EDTA anticoagulant tubes. The samples were then aliquoted into 1.5 mL centrifuge tubes and stored at −20°C. In addition, 66 frozen semen samples were collected from Xinjiang Brown and Brown Swiss bulls used for the artificial insemination of Xinjiang Brown cattle after 1983.

DNA was extracted from the above samples, and the concentration and purity of the obtained genomic DNA were measured using a NanoDrop 2000c spectrophotometer. The OD260/OD280 ratio was 1.7–1.9, indicating good DNA quality. After assessing DNA concentration, purity, and integrity, the samples were stored at −20°C (Ma, 2015).

### 2.2 Sample screening and chip detection

Phenotypically complete Xinjiang Brown cattle were screened from various Xinjiang Brown cattle farms for chip detection. After screening, 403 cows and 82 bulls from four core farms in Xinjiang region were selected. Moreover, we included 174 Xinjiang Brown cows from Xinjiang Uygur Autonomous Region State-owned Urumqi breeding farm, 50 Xinjiang Brown cows from Xinjiang Tianshan Animal Husbandry Bioengineering Co., Ltd. breeding farm, 130 Xinjiang Brown cows from the Tacheng Agriculture and Animal Husbandry Technology Co., Ltd., 49 Xinjiang Brown cows from Yili New Brown breeding farm, 71 bulls and 11 Brown Swiss bulls from Xinjiang Tianshan Animal Husbandry Bioengineering Co., Ltd. Bull breeding station. Chip data for Chinese Holstein cows were obtained from 2,633 animals in Beijing, distributed across 18 farms in the region. All of these animals were detected using the Illumina GeneSeek GGP bovine 150 K chip.

### 2.3 Chip imputation and quality control

A total of 139,376 and 138,892 SNP markers were detected using Xinjiang Brown and Chinese Holstein cattle chip assays, respectively. These data were imputed using Beagle 4.1 software, which infers haplotypes present in the population based on the principle of linkage disequilibrium. To ensure the accuracy of imputation, quality control measures were applied to the chip data.

**FIGURE 1:**
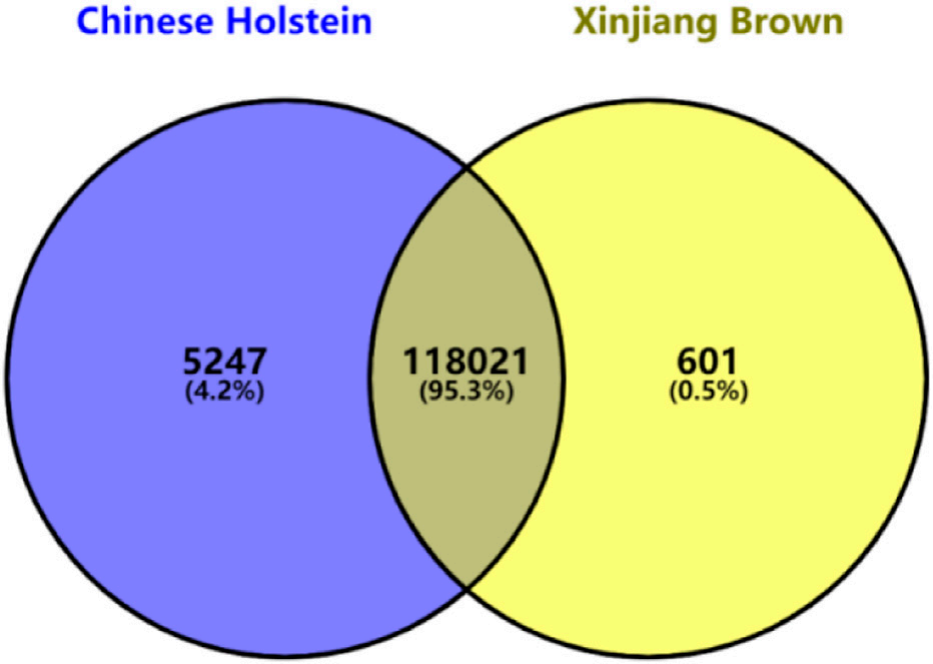
Venn diagram of GeneSeek GGP Bovine 150 k after quality control in Xinjiang Brown cattle and Chinese Holstein cattle.

The quality control criteria were as follows: individuals with a genotyping call rate of <90% were excluded. Only SNPs on chromosomes 1–30 were retained, with an individual genotype missing rate of <10%. SNPs with a minor allele frequency of >0.01 and a Hardy–Weinberg equilibrium *p*-value >1 × 10^−6^ were also included. After quality control using the PLINK software, the SNP genotypes were converted to a 0, 1, and 2 format. Finally, 118,622 and 123,268 SNP markers on the autosomal chromosomes of Xinjiang Brown and Chinese Holstein cattle were retained, respectively. Because the number of SNP markers differed between the two breeds after quality control, an intersection of the SNP markers was taken, which resulted in 118,021 common SNP markers for both breeds (Figure 1).

### 2.4 Genetic structure analysis

#### 2.4.1 Linkage disequilibrium analysis

The.map and. ped files for both breeds were converted to. vcf format using PLINK. The PopLDdecay software was then used to analyze and plot LD decay graphs (https://github.com/BGI-shenzhen/PopLDdecay) (Zhang et al., 2019). The LD metric, r^2^, was calculated for the four populations (Hill, 1974). The mean r^2^ value was computed at various marker distances of 1 Kb to demonstrate the degree of LD decay across different populations.

#### 2.4.2 Population structure analysis

To infer ancestral populations based on the allele frequencies of descendant individuals, an unsupervised algorithm was employed (Consortium et al., 2009). In this study, genome-wide SNP data were used to calculate the population structure for ancestral admixture components with K values of 2–4 using admixture (Alexander et al., 2009). Visualization of the population structure was performed using the R package “pophelper”.

Analysis was conducted using the FastTree software (http://www.microbesonline.org/fasttree/), with the maximum likelihood method adopted for estimation. The Jukes–Cantor + CAT model was used as the default model for nucleotide phylogeny. The credibility of the phylogenetic tree branches was tested using 1,000 bootstrap replicates. Finally, the FigTree software was used for visualization.

#### 2.4.3 Principal component analysis

The Gmatrix package in R was used to calculate the genomic kinship matrix (G-matrix) for Xinjiang Brown and Chinese Holstein cattle. Subsequently, principal component analysis (PCA) was performed using the G-matrix. The first three eigenvectors (PCA1, PCA2, and PCA3) were extracted and used as the horizontal and vertical coordinates for plotting. The contribution rates of the principal components were calculated on the basis of the percentage of eigenvalues. Finally, visualization was performed using the R language.

### 2.5 Multi-breed genomic evaluation using a joint reference population

#### 2.5.1 Phenotypic data processing

The data for Xinjiang Brown cattle include production performance measurement records from 1983 to 2018 and DHI measurement records from 2010 to 2017. The data for Chinese Holstein cattle include DHI measurement records from 2001 to 2019. Milk-related traits, including 305-day milk yield (305dMY), milk fat yield (MFY), milk protein yield (MPY), and somatic cell score (SCS), were obtained through collation (Table 1). There were 7,516 and 93,717 milk trait measurements recorded for Xinjiang Brown and Chinese Holstein cattle, respectively.

**TABLE 1:** The standards for data filtering.

Breed	Character name (unit)^a^	Screening criteria
Xinjiang Brown Cattle milk	305MY(kg)	2,000–1,3000
	MFP(%)	2–7
	MPP(%)	2–7
	SCC(1,000/mL)	0–25,000
Chinese Holstein Cattle milk	305MY(kg)	4,000–15,000
	MFP(%)	2–7
	MPP(%)	2–7
	SCC(1,000/mL)	0–25,000

^a^ 305 dMY: 305 daily milk yield; MFP: milk fat percentage; MPP: milk protein percentage; SCC: somatic cell count.

The pedigree file used to analyze Xinjiang Brown cattle had 16,795 cattle, including 676 breeding bulls. Among these bulls, one had a maximum of 619 offspring, whereas 221 had only one offspring. Among the female adult cattle, 583 had only one offspring, whereas 1,623 had two or more offspring, with a maximum of 12 offspring per individual.

For the Chinese Holstein cattle, the pedigree file used for the analysis contained 6,54,390 individuals, including 11,243 breeding bulls. Among these bulls, one had a maximum of 7,884 offspring, whereas 4,695 had only one offspring. Among the female adult cattle, 1,63,781 had only one offspring, whereas 1,11,912 had two or more offspring, with a maximum of 12 offspring per individual (Table 2).

**TABLE 2:** Data statistics.

Breed	Number	Phenotype animals	Pedigree animals
Xinjiang Brown Cattle	7,516	2,207	16,795
Chinese Holstein Cattle	93,717	48,464	654,390
Total	1,01,233	50,671	6,71,185

#### 2.5.2 Genotype data

Genotype data for 403 female Xinjiang Brown cattle, 71 male Xinjiang Brown cattle, and 11 male Brown Swiss cattle was considered. In addition, 2,100 Chinese Holstein cattle were randomly selected (According to PCA and Admixture results, PLINK software was used to remove the chip data of Chinese Holstein cows that was inconsistent with the large population of Chinese Holstein cows).

#### 2.5.3 Statistical analysis

In this study, the ssGBLUP method was used to construct the H-matrix based on the pedigree and genomic information from Xinjiang Brown and Chinese Holstein cattle. The two-trait model Bayesian approach was used to estimate the variance components and breeding values for each trait.

To investigate the suitable integral ratio of the Chinese Holstein cattle in the joint reference population, a random gradient grouping approach was applied. The population was gradually accumulated in increments of 300 individuals to construct the joint reference population. A control group was established by excluding the phenotypic and genomic information of the Chinese Holstein cattle (Table 3).

**TABLE 3:** Gradient grouping of joint reference group.

Joint reference group	Xinjiang Brown cattle	Chinese holstein cattle
485	485	0
785	485	300
1,085	485	600
1,385	485	900
1,685	485	1,200
1985	485	1,500
2,285	485	1800
2,585	485	2,100

Because of the significant differences in milk production traits between Xinjiang Brown and Chinese Holstein cattle (Zhang et al., 2021), a dual-trait animal model was constructed. In this model, each biological trait was treated individually in the two populations, accounting for potential scale inconsistencies that may arise during breeding value estimation due to standardization across different breeds. The milk production traits (305dMY, MFY, MPY, and SCS) of Xinjiang Brown and Chinese Holstein cattle were considered to be two separate traits. A dual-trait linear model was used to estimate the variance components for milk production traits based on the genomic-pedigree combined relationship matrix, H-matrix. The model is described as follows:

$$\left[\begin{array}{c} y_{1}\\ y_{2} \end{array}\right]=\left[\begin{array}{cc} X_{1} & 0\\ 0 & X_{2} \end{array}\right]\left[\begin{array}{c} \beta_{1}\\ \beta_{2} \end{array}\right]+\left[\begin{array}{cc} Z_{1} & 0\\ 0 & Z_{2} \end{array}\right]\left[\begin{array}{c} a_{1}\\ a_{2} \end{array}\right]+\left[\begin{array}{c} e_{1}\\ e_{2} \end{array}\right]$$

In the formula, $y_{1}$ represents the observation value vector of a certain milk trait for each of Xinjiang Brown cattle, and $y_{2}$ represents Chinese Holstein cattle. $\beta_{1}$ and $\beta_{2}$ represent the fixed effect vectors for the same milk trait of Xinjiang Brown and Chinese Holstein cattle, respectively, including farm effect, calving year effect, calving season effect, and parity effect. The farm effect was divided into 20 levels based on the phenotypic data sources of the farms where the cattle were raised. The calving years of Xinjiang Brown cattle were divided into seven levels based on phenotypic records: 1985–1995, 1996–2000, 2001–2005, 2006–2008, 2009–2011, 2012–2014, and 2015–2018. The calving years of the Chinese Holstein cattle were divided into six levels: 2001–2005, 2006–2008, 2009–2011, 2012–2014, 2015–2018, and 2019. For the parity effect, Xinjiang Brown and Chinese Holstein cattle were classified into six levels: 1, 2, 3, 4, 5, and 6 (including those with more than six calves). The calving season effect of Xinjiang Brown cattle was divided based on the unique climatic conditions of Xinjiang. According to the method of temperature intervals, April and May were considered spring, June, July, and August were considered summer, September was considered autumn, and January, February, March, October, November, and December were considered winter. Conversely, the calving season effect of Chinese Holstein cattle was determined based on the climatic characteristics of Beijing. Following the same method of temperature intervals, March, April, and May were considered spring, June, July, and August were considered summer, September, October, and November were considered autumn, and December, January, and February were considered winter. $a_{1}$ and $a_{2}$ represent the individual additive genetic effect vectors for a certain milk trait of Xinjiang Brown and Chinese Holstein cattle, respectively. $e_{1}$ and $e_{2}$ represent the random residual effect vectors for the same milk trait of Xinjiang Brown and Chinese Holstein cattle, respectively. $X_{i}$ and $Z_{i}$ represent the incidence matrices for the fixed effects and individual random additive genetic effects of the i-th trait, respectively.

Assume $\left[\begin{array}{c} \alpha_{1}\\ \alpha_{2} \end{array}\right]∼N\left(0,H⊗\left[\begin{array}{cc} \sigma_{\alpha_{1}}^{2} & \sigma_{\alpha_{1}\alpha_{2}}\\ \sigma_{\alpha_{2}\alpha_{1}} & \sigma_{\alpha_{2}}^{2} \end{array}\right]\right)$ , $\left[\begin{array}{c} e_{1}\\ e_{2} \end{array}\right]∼N\left(0,I⊗\left[\begin{array}{cc} \sigma_{e_{1}}^{2} & 0\\ 0 & \sigma_{e_{2}}^{2} \end{array}\right]\right)$ , In the aforementioned formula, *H* represents the combined genomic-pedigree relationship matrix. $\sigma_{\alpha_{i}}^{2}$ denotes the additive genetic variance for the i-th breed, while $\sigma_{\alpha_{1}\alpha_{2}}$ represents the covariance between breeds. $\sigma_{e_{i}}^{2}$ stands for the residual variance of the i-th breed. Given that Xinjiang Brown and Chinese Holstein cattle are reared in separate populations, there is no residual covariance between the two groups.

The genetic variance–covariance structure of the ssGBLUP additive genetic effect model is represented by $a∼N\left(0,H\sigma_{a}^{2}\right)$ , where $\sigma_{a}^{2}$ denotes the additive genetic variance. *H*, the pedigree–genome relationship matrix, represents a combination of the pedigree-based additive genetic relationship matrix (A matrix) and the genome-based kinship matrix (G-matrix) (Aguilar et al., 2010; Christensen and Lund, 2010).

The formula used to compute *H* is as follows:

$$H=\left[\begin{array}{cc} A_{11}-A_{12}A_{22}^{-1}A_{21}+A_{12}A_{22}^{-1}{GA}_{21} & A_{12}A_{22}^{-1}G\\ GA_{22}^{-1}A_{21} & G \end{array}\right]$$

Subscripts 1 and 2 in A represent the non-genotyped and genotyped animals in the population, respectively. G represents the genetic relationship matrix. The calculation formula is as follows: $G=\frac{MM^{\prime }}{2\sum_{k=1}^{m}p_{k}\left(1-p_{k}\right)}$ . *M* represents the association matrix for SNP effects, where the elements $\left(0-2p_{j}\right)$ , $\left(1-2p_{j}\right)$ , and $\left(2-2p_{j}\right)$ represent homozygous 11, heterozygous 12 or 21, and homozygous 22 genotypes, respectively. $p_{j}$ represents the minor allele frequency of the jth SNP, and *m* represents the number of markers. $p_{k}$ denotes the allele frequency of the Kth SNP. Therefore, the *H* ^*−1*^ formula is given by $H^{-1}=A^{-1}+\left[\begin{array}{cc} 0 & 0\\ 0 & G^{-1}+A_{22}^{-1} \end{array}\right]$ : where $A^{-1}$ represents the inverse matrix of all pedigree relationships, $G^{-1}$ represents the inverse matrix of genomic kinship relationships, and $A_{22}^{-1}$ represents the inverse matrix of the pedigree relationships for the sequenced individuals. The Bayes method was calculated using the GIBBS1F90 module in the BLUPF90 software along with the Bayes–Gibbs sampling method. In the Bayes method, the total chain length of the samples was 100,000, the burn-in chain length was 10,000, and the thinning interval was 50. The Geweke diagnostic method in POSTGIBBSF90 was used to check the convergence of the Gibbs chain (Zhang et al., 2022).

#### 2.5.4 Calculation of heritability

The calculation formula of heritability is as follows:

$$h^{2}=\frac{\sigma_{a}^{2}}{\sigma_{a}^{2}+\sigma_{e}^{2}}$$

The formula for the standard error of heritability is shown below:

$${SE}^{2}\left(h^{2}\right)=\left[\frac{\sigma_{a}^{2}}{\sigma_{p}^{2}}\right]\left[\frac{Var\left(\sigma_{a}^{2}\right)}{{\left(\sigma_{a}^{2}\right)}^{2}}+\frac{Var\left(\sigma_{p}^{2}\right)}{{\left(\sigma_{p}^{2}\right)}^{2}}-\frac{Cov\left(\sigma_{a}^{2},\sigma_{p}^{2}\right)}{\sigma_{a}^{2}\sigma_{p}^{2}}\right]$$

where $h^{2}$ is heritability, ${SE}^{2}\left(h^{2}\right)\text{ is the standard error of heritability},\sigma_{a}^{2}$ is the additive genetic variance, $\sigma_{e}^{2}$ is the residual variance. $\sigma_{p}^{2}$ is the overall phenotypic variance, $\sigma_{p}^{2}=\sigma_{a}^{2}+\sigma_{e}^{2}$ .

#### 2.5.5 Verification of the reliability of genomic breeding values

To verify the accuracy of the estimated genomic breeding values for the joint reference populations, 50 offspring individuals born in the past 4 years from 485 genotyped Xinjiang Brown cattle served as a validation group. Genomic predictions were performed in two groups: with and without excluding the phenotypic data of the validation group. This resulted in 16 sets of genetic parameters and genomic estimated breeding values for each milk trait. By comparing the prediction accuracy, estimation bias, and inflation coefficient of the validation group, the optimal number of joint reference populations was determined.

To calculate the prediction accuracy of the genomic estimated breeding values, the correlation coefficient between the genomic breeding values calculated with the phenotypic data of the validation group and those calculated without these data was used to measure the accuracy of estimating genomic breeding values for different joint reference populations. The formula is as follows: $R_{\mathrm{GEBV}}=\text{Cor}\left({\mathrm{TBV}}^{*},\text{GEBV}\right)$ , where $R^{2}$ represents reliability or the square of accuracy.

Meanwhile, the regression of ${\mathrm{TBV}}^{*}$ on GEBV is calculated using the formula ${\mathrm{TBV}}^{*}=b_{0}+b_{1}\mathrm{GEBV}$ , where the regression coefficient $b_{1}$ is the inflation coefficient, and the intercept $b_{0}$ is the estimation bias (Legarra and Reverter, 2019).

$$b_{1}=\frac{cov\left(GEBV,y\right)}{var\left(y\right)}=\frac{var\left(GEBV\right)}{var\left(y\right)}<1$$

$$b_{1}=\frac{Var\left(GEBV\right)}{Var\left(y\right)}=\frac{Var\left(GEBV\right)Var\left(a\right)}{Var\left(a\right)Var\left(y\right)}=r_{GEBV}^{2}r_{y}^{2}\ll 1$$

In this context, $b_{1}$ represents the regression coefficient, which has the following implications: when $b_{1}<1$ , GEBV is inflated, indicating that Var (GEBV) is too large. This means that in the genomic breeding values, the good ones are even better, and the bad ones are even worse. Conversely, when $b_{1}>1$ , GEBV is deflated, suggesting that Var (GEBV) is too small. This indicates that the genomic breeding values are smaller than the true values and are contracted toward the middle.

## 3 Results

### 3.1 Genetic structure

#### 3.1.1 Linkage disequilibrium analysis

**FIGURE 2:**
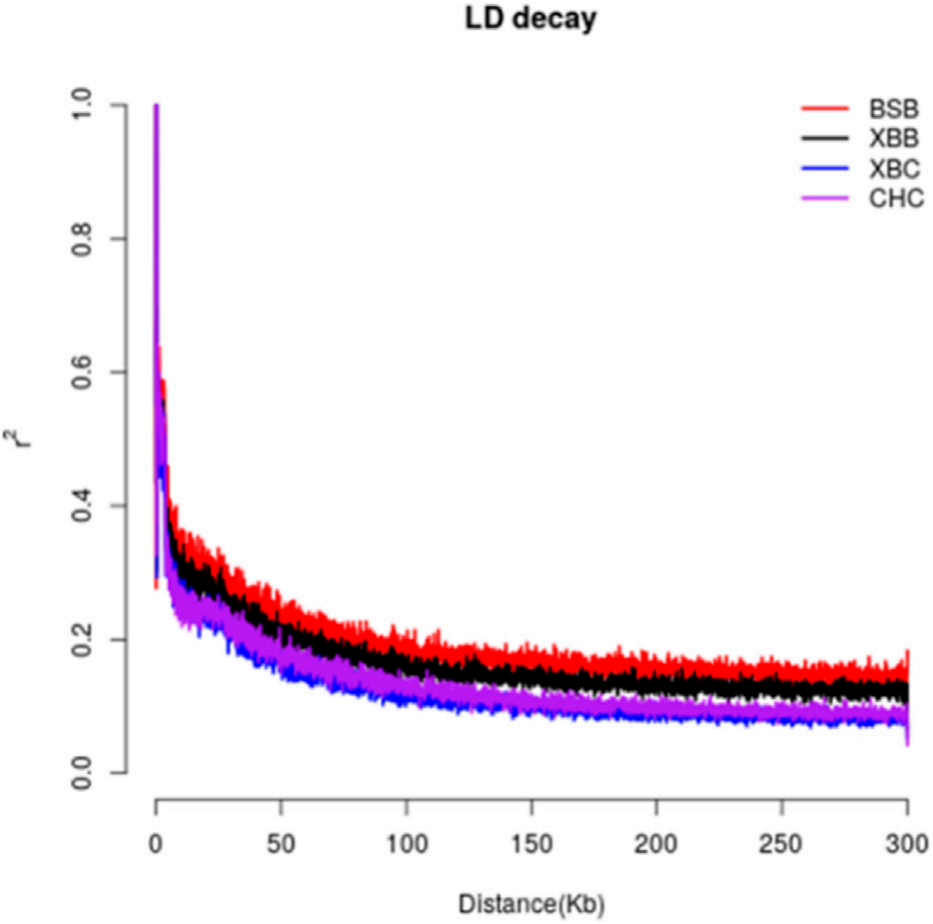
LD decay of Xinjiang Brown cattle and Chinese Holstein cow. BSB is Brown Swiss Bull, CHC is Chinese Holstein cow, XBB is Xinjiang Brown Bull, XBC is Xinjiang Brown Cow.

Linkage disequilibrium analysis was conducted between the two breeds by calculating the linkage disequilibrium coefficients for the two loci and plotting the LD decay graph (Figure 2). The graph shows that the average LD coefficients for Xinjiang Brown cows, Xinjiang Brown bulls, Brown Swiss bulls, and Chinese Holstein cows at a genomic distance of 50 kb were approximately 0.2, 0.25, 0.3, and 0.35, respectively, indicating a gradual increase. Noteworthy, the decay rates of the LD coefficients vary among different populations. Among the breeds, Brown Swiss bulls exhibited the slowest LD decay at 0–40 kb, whereas the Chinese Holstein cows exhibited the fastest LD decay. However, in the range of 40–300 kb, Xinjiang Brown cows exhibited the fastest LD decay, with a decay rate order of Xinjiang Brown cows > Chinese Holstein cows > Xinjiang Brown bulls > Brown Swiss bulls.

#### 3.1.2 Population structure analysis

**FIGURE 3:**
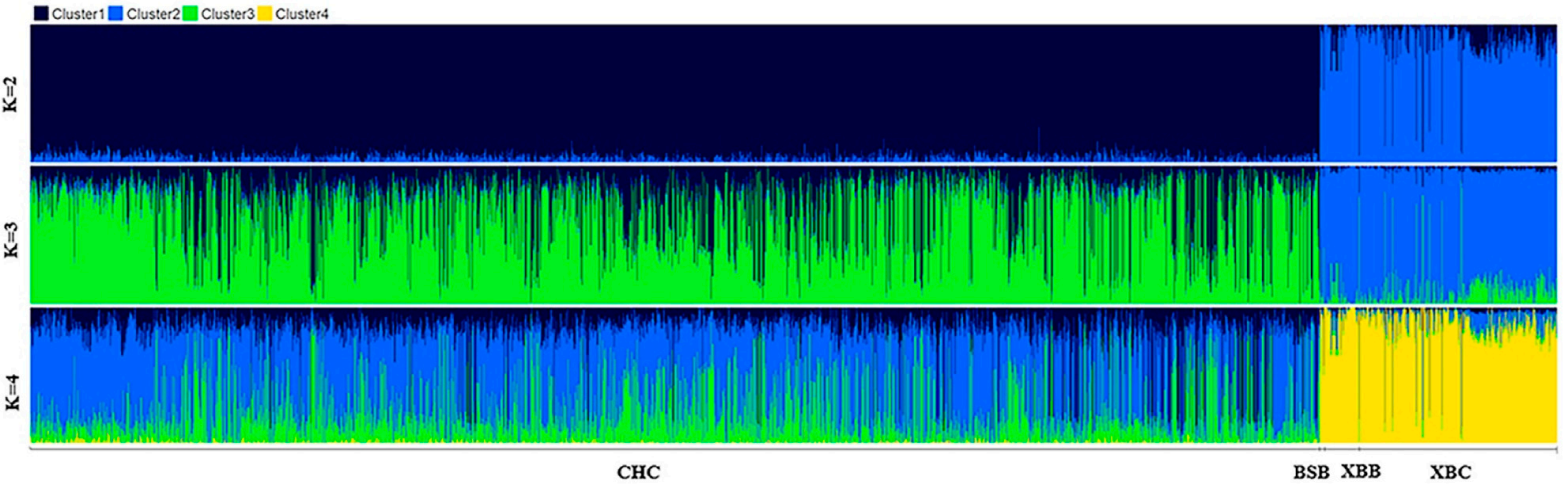
Analysis chart of population structure in Xinjiang Brown cattle and Chinese Holstein cow. BSB is Brown Swiss Bull, CHC is Chinese Holstein cow, XBB is Xinjiang Brown Bull, XBC is Xinjiang Brown Cow.

**FIGURE 4:**
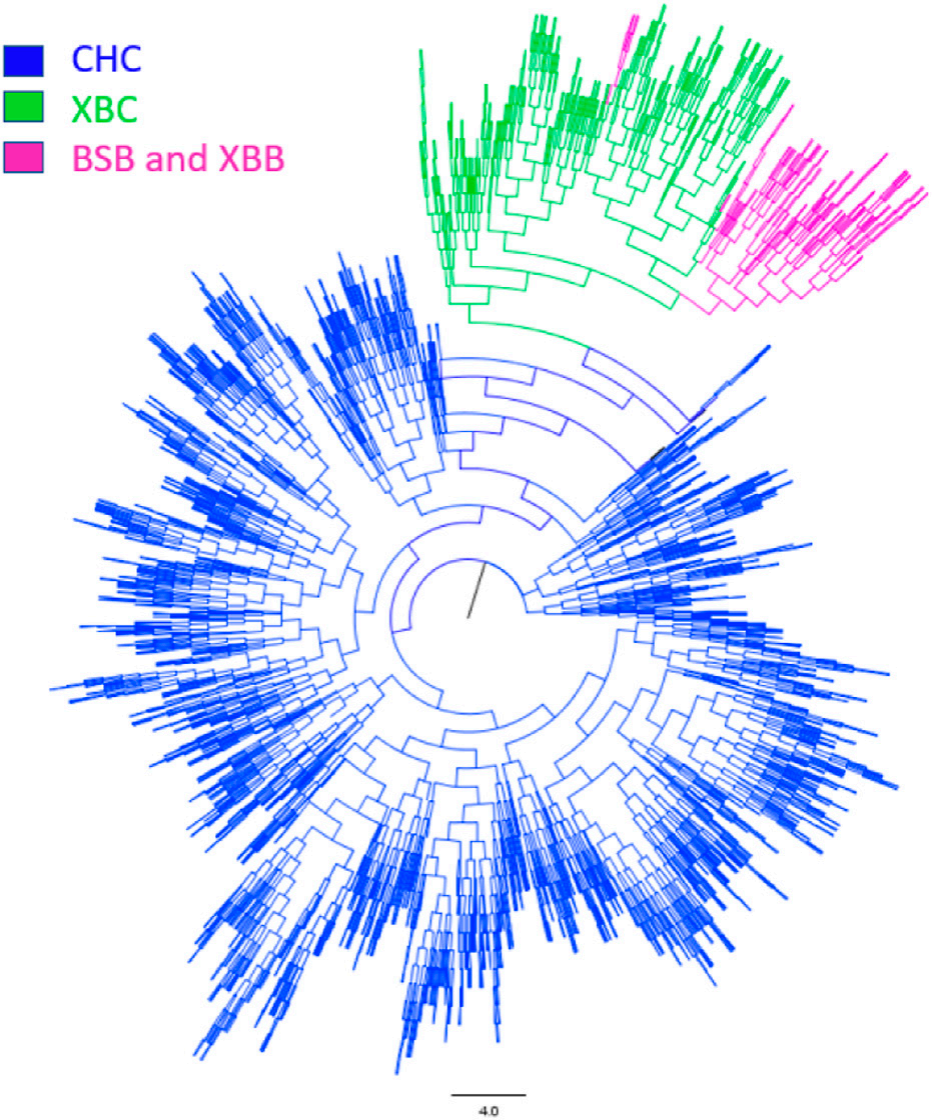
Phylogenetic tree of Xinjiang Brown cattle and Chinese Holstein cow. BSB is Brown Swiss Bull, CHC is Chinese Holstein cow, XBB is Xinjiang Brown Bull, XBC is Xinjiang Brown Cow.

To further investigate the genetic components of Xinjiang Brown and Chinese Holstein cows, population structure and phylogenetic tree analyses were conducted. As shown in Figure 3, when the number of ancestral populations K = 2, there was a clear distinction in the genetic structure among Xinjiang Brown cows, Xinjiang Brown bulls, Brown Swiss bulls, and Chinese Holstein cows. However, the genetic structure within each group differed insignificantly. As shown in Figure 4, Xinjiang Brown cows, Xinjiang Brown bulls, and Brown Swiss bulls were clustered, whereas the Chinese Holstein cows were clustered separately. In addition, Xinjiang Brown cows, Xinjiang Brown bulls, and Brown Swiss bulls appear at the end of a certain branch within the Chinese Holstein population.

#### 3.1.3 Genetic relatedness between Xinjiang Brown and Chinese Holstein cows

**FIGURE 5:**
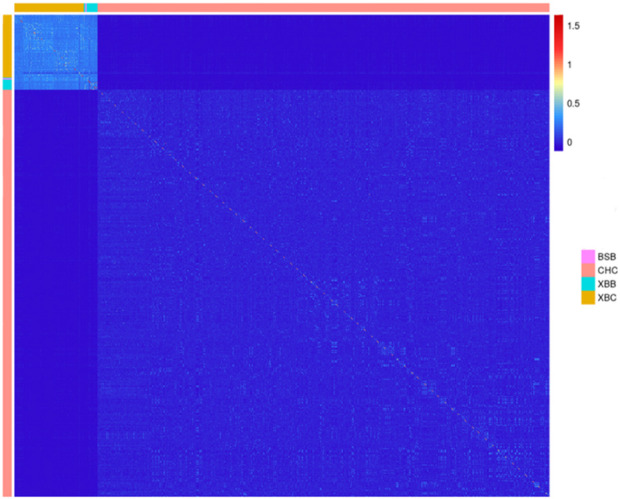
Genomic relationship matrix of Xinjiang Brown cattle and Chinese Holstein cow. BSB is Brown Swiss Bull, CHC is Chinese Holstein cow, XBB is Xinjiang Brown Bull, XBC is Xinjiang Brown Cow.

Using the SNP genotyping information from 403 Xinjiang Brown cows, 71 Xinjiang Brown bulls, 11 Brown Swiss bulls, and 2,633 Chinese Holstein cows, a G-matrix was constructed. Figure 5 was then generated on the basis of the actual genetic relatedness among individuals in the G-matrix. Figure 5 shows that the kinship coefficients among Xinjiang Brown cows, Xinjiang Brown bulls, and Brown Swiss bulls populations were approximately 0.5, which is significantly higher than those among individuals within the Chinese Holstein cow population. The kinship coefficients between Xinjiang Brown cows, Xinjiang Brown bulls, Brown Swiss bulls, and Chinese Holstein cows populations tend toward 0.

#### 3.1.4 PCA

**FIGURE 6:**
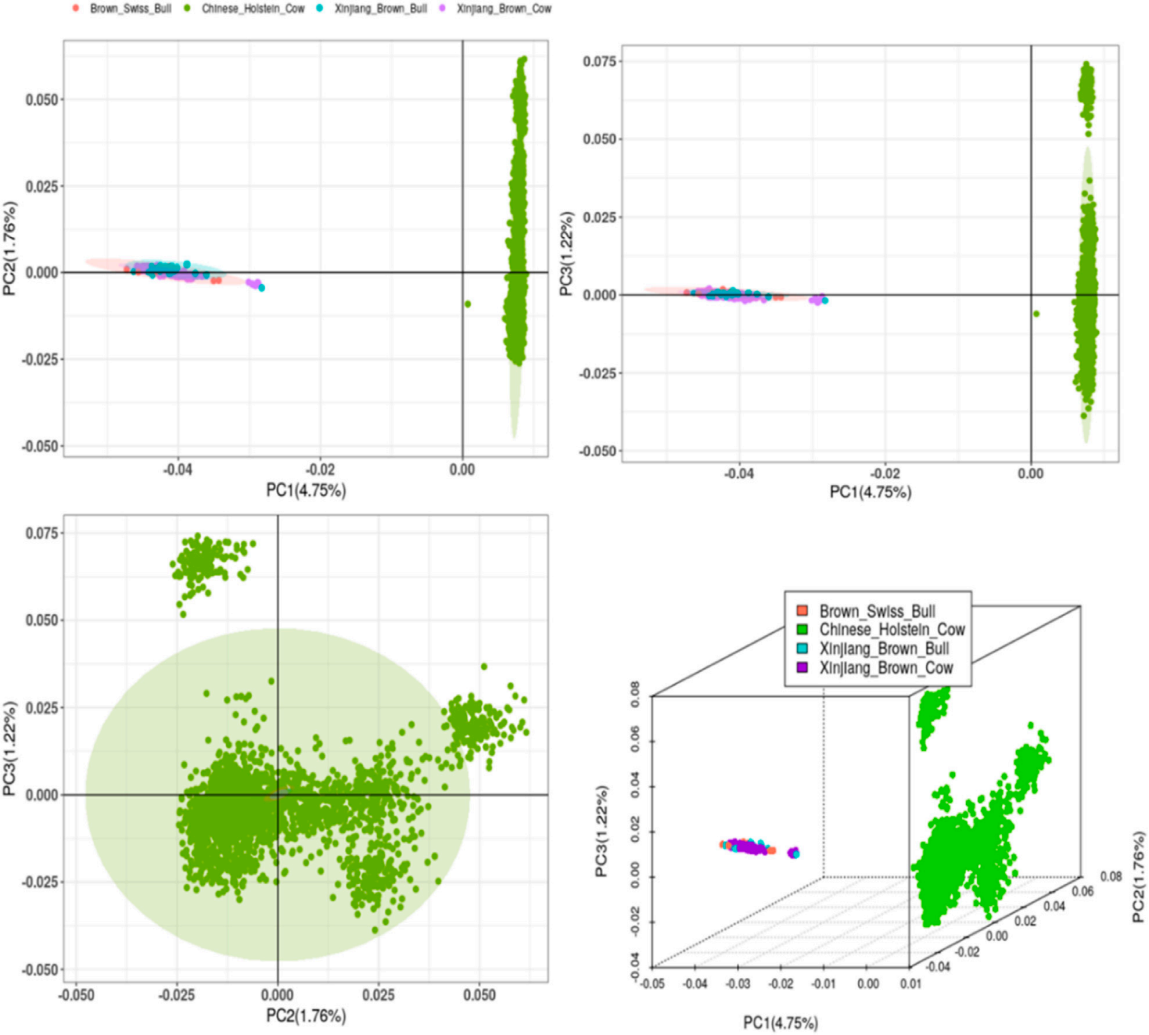
Principal Component Analysis of Xinjiang Brown Cattle and Chinese Holstein cow.

**FIGURE 7:**
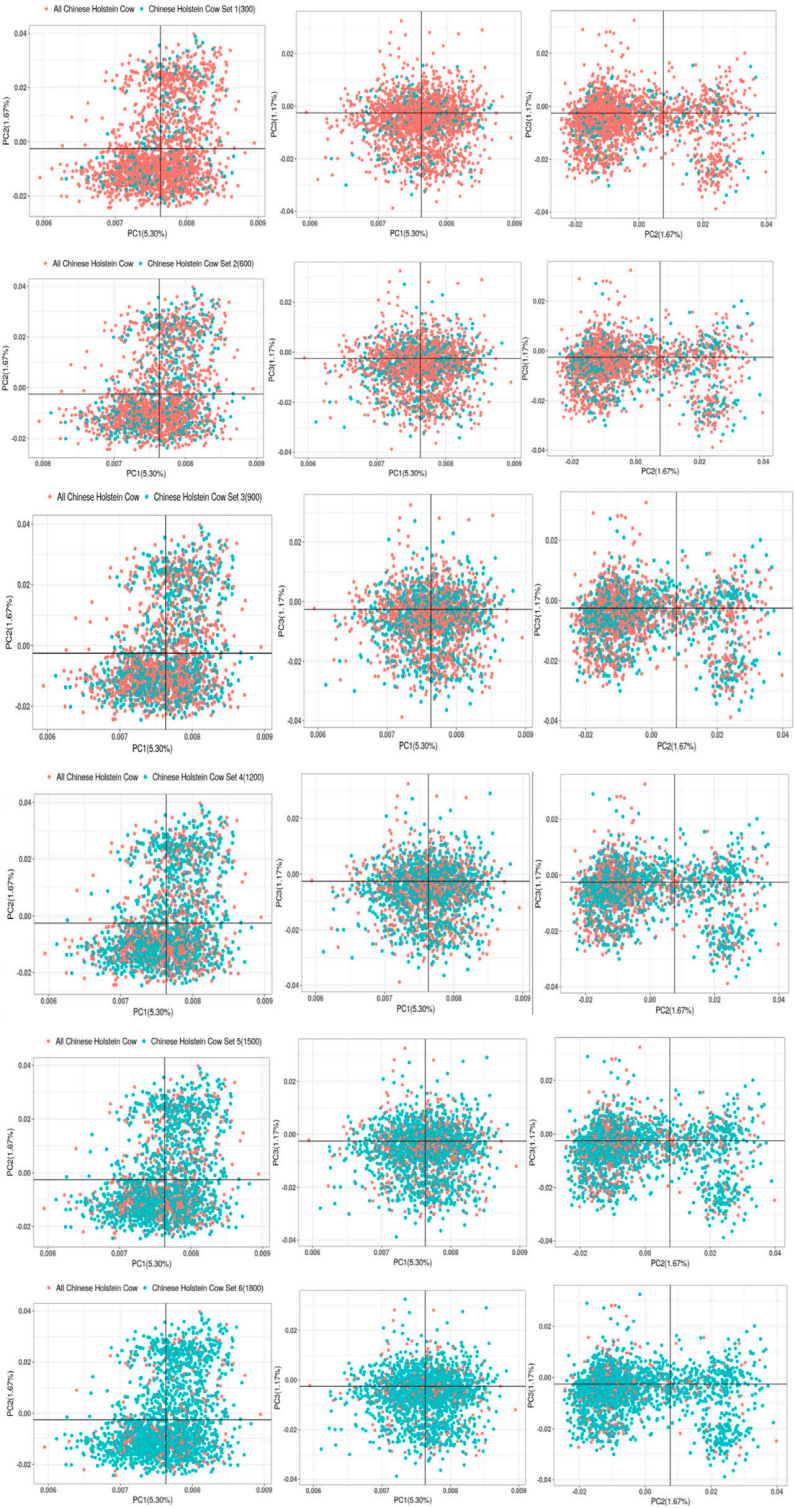
Gradient grouping of PCA analysis in joint reference group.

PCA was performed using the genomic kinship relationship matrix (G-matrix) among individuals from the two breeds (Figures 2–7). The results revealed that the first principal component (PC1, accounting for 4.75%) separated Xinjiang Brown cows, Xinjiang Brown bulls, Brown Swiss bulls, and Chinese Holstein cows into distinct groups. Specifically, Xinjiang Brown cows, Xinjiang Brown bulls, and Brown Swiss bulls were closely clustered. The second principal component (PC2, accounting for 1.76%) could not distinguish between Xinjiang Brown cows, Xinjiang Brown bulls, and Brown Swiss bulls; however, it separated the Chinese Holstein cows into two distinct groups. The third principal component (PC3, accounting for 1.22%) further distinguished the Chinese Holstein cows into two groups.

### 3.2 Multi-breed genomic evaluation using a joint reference population

#### 3.2.1 Descriptive statistics of the dairy traits of Xinjiang brown and Chinese Holstein cattle

Table 4 lists the statistics, including sample size, minimum value, maximum value, mean, standard deviation, and coefficient of variation of the observed dairy traits of Xinjiang Brown and Chinese Holstein cattle. 305dMY, MFY, MPY, and SCS between Xinjiang Brown and Chinese Holstein cattle differed significantly.

**TABLE 4:** Description of milk traits in Xinjiang Brown Cattle and Chinese Holstein Cattle.

Breed	Trait^a^	Number	Minimum	Maximum	Average	SD	CV(%)
Xinjiang Brown	305dMY/kg	7,515	814	8,444	4126.49	1405.71	34.07
	MFY/kg	2,655	21.6	431.55	168.53	68.29	40.52
	MPY/kg	2,655	20.3	302.72	143.71	51.42	35.78
	SCS	2,655	−2.05	10.95	4.98	2.16	43.37
Chinese Holstein	305dMY/kg	89,350	4001	15,000	10116.68	2045.07	20.21
	MFY/kg	89,350	86.30	1019.21	398.52	117.43	29.47
	MPY/kg	89,350	87.72	951.76	329.92	74.40	22.55
	SCS	89,350	−3.65	9.65	3.25	1.81	55.66

^a^ 305 dMY: 305 daily milk yield; MFY: milk fat yield; MPY: milk protein yield; SCS: somatic cell score. SD: Standard deviation.

#### 3.2.2 Random grouping of the joint reference group

The chip data of 2,633 Chinese Holstein cows were screened to eliminate individuals with distant kinship within the Chinese Holstein cattle population, leaving 2,271 genotyped Chinese Holstein cows. Among these, 2,100 cows were randomly selected as the total population of Chinese Holstein cows to be included in the joint reference group. Random equal-sized groupings were then performed on 2,100 genotyped Chinese Holstein cows, resulting in subsets with different numbers of cows. As shown in Figure 7, the distribution of the subsets in the total population was relatively scattered for the Chinese Holstein cows added to the joint reference group.

#### 3.2.3 Estimation of genetic parameters of dairy traits in the joint reference group

As shown in Table 5, before the inclusion of the Chinese Holstein population, the heritability of 305dMY was 0.204 without excluding the phenotypic data of 50 genotype Xinjiang Brown cows, which decreased to 0.203 after data exclusion. When varying numbers of Chinese Holstein cows were incorporated into the joint reference group, the heritability of 305dMY in Xinjiang Brown cows was 0.137–0.249 without excluding the phenotypic data of the 50 genotype cows. However, after excluding these data, the heritability of 305dMY in Xinjiang Brown cows was adjusted to 0.169–0.254.

**TABLE 5:** Genetic parameter estimation of 305dMY in joint reference population based on ssGBLUP.

Joint reference population	Xinjiang Brown	Chinese holstein	Breed	All data			All data-50		
				σ_a_^2^(SE)	σ_e_^2^(SE)	h^2^(SE)	σ_a_^2^(SE)	σ_e_^2^(SE)	h^2^(SE)
485	485	0	Xinjiang Brown	235,810	925,050	0.204	237,340	931,960	0.203
				(23,428)	(19,516)	(0.018)	(23,720)	(19,687)	(0.018)
785	485	300	Xinjiang Brown	395,790	2,495,000	0.137	605,190	2,378,800	0.203
				(256,700)	(292,170)	(0.081)	(236,900)	(272,020)	(0.072)
785	485	300	Chinese Holstein	245,370	923,400	0.21	245,710	930,680	0.209
				(24,660)	(19,243)	(0.019)	(24,693)	(19,285)	(0.019)
1,085	485	600	Xinjiang Brown	768,210	2,324,100	0.249	785,140	2,313,700	0.254
				(205,040)	(181,580)	(0.057)	(192,460)	(180,370)	(0.054)
1,085	485	600	Chinese Holstein	246,200	922,650	0.211	246,250	930,390	0.21
				(24,449)	(18,966)	(0.019)	(24,649)	(19,962)	(0.019)
1,385	485	900	Xinjiang Brown	635,210	2,316,300	0.216	544,100	2,363,400	0.188
				(125,820)	(129,040)	(0.038)	(122,830)	(136,000)	(0.039)
1,385	485	900	Chinese Holstein	243,990	923,970	0.209	249,440	929,320	0.212
				(24,038)	(19,290)	(0.018)	(24,562)	(19,131)	(0.018)
1,685	485	1,200	Xinjiang Brown	591,430	2,562,300	0.188	523,940	2,586,200	0.169
				(130,800)	(123,390)	(0.037)	(75,741)	(110,240)	(0.023)
1,685	485	1,200	Chinese Holstein	243,880	923,950	0.209	253,520	926,610	0.215
				(24,708)	(19,457)	(0.019)	(23,774)	(19,612)	(0.018)
1985	485	1,500	Xinjiang Brown	610,630	2,556,100	0.193	582,790	2,571,100	0.185
				(107,850)	(101,890)	(0.031)	(119,430)	(105,660)	(0.035)
1985	485	1,500	Chinese Holstein	242,740	923,660	0.209	246,930	929,650	0.21
				(24,064)	(19,028)	(0.018)	(24,018)	(19,202)	(0.018)
2,285	485	1800	Xinjiang Brown	629,300	2,573,000	0.197	637,850	2,569,600	0.199
				(97,901)	(92,596)	(0.028)	(102,810)	(94,655)	(0.029)
2,285	485	1800	Chinese Holstein	244,750	923,220	0.21	246,040	930,360	0.21
				(23,787)	(18,710)	(0.018)	(25,235)	(19,941)	(0.019)
2,585	485	2,100	Xinjiang Brown	654,250	2,513,800	0.207	642,470	2,520,000	0.204
				(91,086)	(85,262)	(0.026)	(91,676)	(83,306)	(0.026)
2,585	485	2,100	Chinese Holstein	242,120	924,250	0.208	247,120	929,710	0.21
				(23,562)	(19,207)	(0.018)	(25,612)	(19,792)	(0.019)

Note: “All data” refers to the calculation results without excluding the phenotypic data of the 50 validation animals; “All data-50” refers to the calculation results after excluding the phenotypic data of the 50 validation animals. σ^2^_a_ = additive genetic variance; σ^2^_E_ = residual variance; *h* ^*2*^ = heritability; SE, standard error.

As shown in Table 6, without the inclusion of Chinese Holstein cows, the heritability of MFY, was 0.07 when the phenotypic data of 50 genotype Xinjiang Brown cows were included, and it was 0.073 when the phenotypic data were excluded. When different numbers of Chinese Holstein cows were added to the reference population, the MFY, heritability of Xinjiang Brown cows was 0.073–0.086 when the phenotypic data of 50 genotype Xinjiang Brown cows were included, and it was 0.057–0.085 when the phenotypic data were excluded.

**TABLE 6:** Genetic parameter estimation of MFY in joint reference population based on ssGBLUP.

Joint reference population	Xinjiang Brown	Chinese holstein	Breed	All data			All data-50		
				σ_a_^2^(SE)	σ_e_^2^(SE)	h^2^(SE)	σ_a_^2^(SE)	σ_e_^2^(SE)	h^2^(SE)
485	485	0	Xinjiang Brown	211.01	2843.6	0.07	225.03	2879.9	0.073
				(64.235)	(94.068)	(0.021)	(67.683)	(97.71)	(0.022)
785	485	300	Xinjiang Brown	260.07	2820.2	0.085	261.65	2860.5	0.084
				(70.93)	(92.423)	(0.023)	(73.108)	(97.286)	(0.023)
785	485	300	Chinese Holstein	667.02	10,151	0.062	1,091	9870.2	0.1
				(628.76)	(1056.8)	(0.056)	(663.79)	(1014.2)	(0.057)
1,085	485	600	Xinjiang Brown	261.42	2821.7	0.085	260.63	2863.3	0.084
				(72.708)	(92.754)	(0.023)	(68.761)	(96.963)	(0.022)
1,085	485	600	Chinese Holstein	1,248	9922.6	0.112	1594.4	9682.6	0.142
				(522.17)	(703.23)	(0.045)	(544.89)	(678.09)	(0.046)
1,385	485	900	Xinjiang Brown	262.8	2819.4	0.086	251.19	2865.4	0.081
				(66.884)	(93.427)	(0.021)	(77.111)	(97.509)	(0.024)
1,385	485	900	Chinese Holstein	1015.3	10,726	0.087	734.34	10,921	0.064
				(414.44)	(553.17)	(0.034)	(511.15)	(601.11)	(0.043)
1,685	485	1,200	Xinjiang Brown	256.98	2821.5	0.084	263.17	2860.9	0.085
				(67.492)	(94.732)	(0.022)	(80.748)	(97.523)	(0.025)
1,685	485	1,200	Chinese Holstein	715.82	10,683	0.063	838.62	10,581	0.074
				(372.83)	(497.08)	(0.032)	(339.32)	(467.88)	(0.029)
1985	485	1,500	Xinjiang Brown	253.46	2820.4	0.083	249.42	2866.8	0.081
				(58.914)	(91.339)	(0.019)	(75.035)	(96.106)	(0.024)
1985	485	1,500	Chinese Holstein	629.08	10,465	0.057	785.9	10,349	0.071
				(317.84)	(417.27)	(0.028)	(285.16)	(399.72)	(0.025)
2,285	485	1800	Xinjiang Brown	266.36	2818.9	0.087	242.33	2871.7	0.078
				(73.755)	(97.11)	(0.023)	(61.402)	(94.469)	(0.02)
2,285	485	1800	Chinese Holstein	717.61	10,529	0.064	754.32	10,503	0.068
				(239.88)	(360.06)	(0.021)	(266.09)	(364.18)	(0.024)
2,585	485	2,100	Xinjiang Brown	223.39	2843.5	0.073	174.8	2,917	0.057
				(67.122)	(91.748)	(0.022)	(74.579)	(101.76)	(0.024)
2,585	485	2,100	Chinese Holstein	1037.2	10,437	0.091	1139.2	10,376	0.099
				(256.92)	(336.28)	(0.022)	(249.42)	(329.61)	(0.021)

Note: “All data” refers to the calculation results without excluding the phenotypic data of the 50 validation animals; “All data-50” refers to the calculation results after excluding the phenotypic data of the 50 validation animals. σ^2^_a_ = additive genetic variance; σ^2^_E_ = residual variance; *h* ^*2*^ = heritability; SE, standard error.

As shown in Table 7, without the inclusion of the Chinese Holstein cows, the heritability of MPY, was 0.143 when the phenotypic data of 50 genotyped Xinjiang Brown cows were included, and it was 0.145 when the phenotypic data were excluded. When different numbers of Chinese Holstein cows were added to the reference population, the MPY, heritability of Xinjiang Brown cows was 0.123–0.158 when the phenotypic data of the 50 genotyped Xinjiang Brown cows were included, and it was 0.0142–0.174 when the phenotypic data were excluded.

**TABLE 7:** Genetic parameter estimation of MPY in joint reference population based on ssGBLUP.

Joint reference population	Xinjiang Brown	Chinese holstein	Breed	All data			All data-50		
				σ_a_^2^(SE)	σ_e_^2^(SE)	h^2^(SE)	σ_a_^2^(SE)	σ_e_^2^(SE)	h^2^(SE)
485	485	0	Xinjiang Brown	238.44	1429.4	0.143	246.05	1455.9	0.145
				(46.431)	(48.997)	(0.026)	(47.902)	(50.543)	(0.027)
785	485	300	Xinjiang Brown	253.09	1423.1	0.151	267.32	1446.7	0.156
				(50.485)	(49.704)	(0.028)	(48.616)	(48.525)	(0.027)
785	485	300	Chinese Holstein	463.33	3956.7	0.105	451.38	3955.5	0.103
				(477.27)	(464.24)	(0.1)	(382.5)	(461.92)	(0.081)
1,085	485	600	Xinjiang Brown	261.17	1419.4	0.156	264.41	1450.2	0.155
				(48.804)	(47.058)	(0.027)	(51.41)	(49.689)	(0.028)
1,085	485	600	Chinese Holstein	786.7	3971.3	0.166	828.05	3935.7	0.174
				(310.95)	(308.68)	(0.061)	(280.6)	(277.55)	(0.053)
1,385	485	900	Xinjiang Brown	264.73	1419.4	0.158	265.12	1450.5	0.155
				(51.675)	(48.283)	(0.029)	(47.87)	(49.206)	(0.026)
1,385	485	900	Chinese Holstein	544.43	3886.4	0.123	593.77	3858.1	0.134
				(188.04)	(222.02)	(0.041)	(172.04)	(204.45)	(0.036)
1,685	485	1,200	Xinjiang Brown	255.49	1423.7	0.153	265.08	1447.8	0.155
				(49.583)	(48.699)	(0.028)	(50.392)	(52.497)	(0.028)
1,685	485	1,200	Chinese Holstein	576.71	3958.4	0.128	560.76	3962.6	0.124
				(140.64)	(175.24)	(0.03)	(149.43)	(183.38)	(0.032)
1985	485	1,500	Xinjiang Brown	257.41	1422.2	0.154	250.87	1457.3	0.147
				(48.761)	(48.203)	(0.027)	(52.614)	(52.575)	(0.029)
1985	485	1,500	Chinese Holstein	563.84	3980.9	0.125	576.8	3988.6	0.127
				(136.03)	(154.87)	(0.029)	(118.89)	(155.55)	(0.025)
2,285	485	1800	Xinjiang Brown	249.04	1424.2	0.149	261.17	1451.4	0.153
				(48.875)	(48.052)	(0.028)	(48.144)	(49.9)	(0.026)
2,285	485	1800	Chinese Holstein	653.65	3916.5	0.144	645.01	3918.3	0.142
				(115.42)	(138.07)	(0.024)	(128.01)	(138.72)	(0.027)
2,585	485	2,100	Xinjiang Brown	246.36	1427.5	0.148	243.18	1458.6	0.143
				(41.863)	(47.265)	(0.024)	(52.403)	(51)	(0.029)
2,585	485	2,100	Chinese Holstein	7,112.74	3795.6	0.158	717.43	3796.2	0.159
				(118.26)	(126.31)	(0.025)	(116.58)	(119.39)	(0.024)

Note: “All data” refers to the calculation results without excluding the phenotypic data of the 50 validation animals; “All data-50” refers to the calculation results after excluding the phenotypic data of the 50 validation animals. σ^2^_a_ = additive genetic variance; σ^2^_E_ = residual variance; *h* ^*2*^ = heritability; SE, standard error.

Table 8 shows that without the inclusion of the Chinese Holstein cow population, the heritability of SCS was 0.042 when the phenotypic data of the 50 genotyped Xinjiang Brown cows were included and 0.043 when the phenotypic data were excluded. After adding different numbers of the Chinese Holstein cows to the joint reference population, the SCS heritability for Xinjiang Brown cows was 0.02–0.062 when the phenotypic data of the 50 genotyped Xinjiang Brown cows were included. When the phenotypic data were excluded, the SCS heritability for Xinjiang Brown cows was 0.015–0.081.

**TABLE 8:** Genetic parameter estimation of SCS in joint reference population based on ssGBLUP.

Joint reference population	Xinjiang Brown	Chinese holstein	Breed	All data			All data-50		
				σ_a_^2^(SE)	σ_e_^2^(SE)	h^2^(SE)	σ_a_^2^(SE)	σ_e_^2^(SE)	h^2^(SE)
485	485	0	Xinjiang Brown	0.174	4.043	0.042	0.18	4.015	0.043
				(0.073)	(0.127)	(0.017)	(0.079)	(0.131)	(0.019)
785	485	300	Xinjiang Brown	0.143	2.721	0.05	0.235	2.687	0.081
				(0.073)	(0.124)	(0.045)	(0.204)	(0.271)	(0.065)
785	485	300	Chinese Holstein	0.216	4.026	0.051	0.184	4.029	0.044
				(0.077)	(0.124)	(0.018)	(0.089)	(0.131)	(0.021)
1,085	485	600	Xinjiang Brown	0.159	2.718	0.056	0.106	2.76	0.037
				(0.107)	(0.18)	(0.037)	(0.113)	(0.181)	(0.039)
1,085	485	600	Chinese Holstein	0.213	4.033	0.051	0.234	3.99	0.056
				(0.802)	(0.129)	(0.019)	(0.855)	(0.127)	(0.02)
1,385	485	900	Xinjiang Brown	0.177	2.68	0.062	0.125	2.711	0.044
				(0.088)	(0.133)	(0.03)	(0.083)	(0.138)	(0.029)
1,385	485	900	Chinese Holstein	0.199	4.039	0.047	0.232	3.992	0.055
				(0.073)	(0.123)	(0.017)	(0.081)	(0.127)	(0.019)
1,685	485	1,200	Xinjiang Brown	0.139	2.891	0.046	0.134	2.896	0.045
				(0.039)	(0.112)	(0.013)	(0.055)	(0.114)	(0.018)
1,685	485	1,200	Chinese Holstein	0.218	4.027	0.052	0.2	4.01	0.048
				(0.058)	(0.123)	(0.014)	(0.071)	(0.127)	(0.017)
1985	485	1,500	Xinjiang Brown	0.09	2.899	0.03	0.918	2.896	0.031
				(0.046)	(0.101)	(0.016)	(0.463)	(0.104)	(0.016)
1985	485	1,500	Chinese Holstein	0.226	4.021	0.054	0.229	3.988	0.055
				(0.078)	(0.124)	(0.018)	(0.077)	(0.125)	(0.018)
2,285	485	1800	Xinjiang Brown	0.058	2.923	0.02	0.074	2.913	0.025
				(0.043)	(0.095)	(0.015)	(0.042)	(0.091)	(0.014)
2,285	485	1800	Chinese Holstein	0.208	4.03	0.049	0.201	4.011	0.048
				(0.069)	(0.122)	(0.016)	(0.089)	(0.13)	(0.021)
2,585	485	2,100	Xinjiang Brown	0.057	2.863	0.02	0.044	2.868	0.015
				(0.044)	(0.082)	(0.015)	(0.032)	(0.084)	(0.011)
2,585	485	2,100	Chinese Holstein	0.218	4.024	0.052	0.197	4.014	0.047
				(0.082)	(0.129)	(0.019)	(0.099)	(0.133)	(0.023)

Note: “All data” refers to the calculation results without excluding the phenotypic data of the 50 validation animals; “A ll data-50” refers to the calculation results after excluding the phenotypic data of the 50 validation animals. σ^2^_a_ = additive genetic variance; σ^2^_E_ = residual variance; *h* ^*2*^ = heritability; SE, standard error.

#### 3.2.4 Verification of the genomic breeding value reliability

As shown in Table 9, when different numbers of Chinese Holstein cows were added to the joint reference population, the reliability of the total population genomic breeding values for 305dMY of Xinjiang Brown cows was 0.142–0.340, with a regression coefficient of 0.129–0.312. The reliability of the genomic breeding values for the validation population was −0.033–0.087, with a regression coefficient of −0.064–0.056.

**TABLE 9:** Genetic parameter estimation of 305dMY in joint reference population based on ssGBLUP.

Joint reference population	Xinjiang Brown	Chinese holstein	Total population			Validation population		
			R^2^_GEBV_	b_0_	b_1_	R^2^_GEBV_	b_0_	b_1_
485	485	0	0.172	−66.82	0.171	0.225	−101.864	0.42
785	485	300	0.272	−27.443	0.271	−0.041	−36.453	−0.034
1,085	485	600	0.196	−25.215	0.201	0.087	−38.312	0.056
1,385	485	900	0.142	−25.038	0.129	0.04	−38.802	0.027
1,685	485	1,200	0.340	−12.308	0.312	−0.061	−64.426	−0.037
1985	485	1,500	0.245	−12.074	0.242	−0.083	−72.544	−0.064
2,285	485	1800	0.302	−14.355	0.291	−0.033	−71.878	−0.024
2,585	485	2,100	0.205	−18.233	0.257	−0.147	−80.443	−0.13

As shown in Table 10, when different numbers of the Chinese Holstein cows were added to the joint reference population, the reliability of the total population genomic breeding values for MFY, of Xinjiang Brown cows was 0.263–0.424, with a regression coefficient of 0.296–0.437. The reliability of the genomic breeding values for the validation population was −0.149–0.138, with a regression coefficient of −0.192–0.137.

**TABLE 10:** Genetic parameter estimation of MFY in joint reference population based on ssGBLUP.

Joint reference population	Xinjiang Brown	Chinese holstein	Total population			Validation population		
			R^2^_GEBV_	b_0_	b_1_	R^2^_GEBV_	b_0_	b_1_
485	485	0	0.300	−1.492	0.296	−0.141	−0.776	−0.083
785	485	300	0.414	−0.332	0.437	0.035	−2.114	0.038
1,085	485	600	0.404	−0.236	0.413	0.138	−2.734	0.137
1,385	485	900	0.374	−0.189	0.41	−0.065	−3.829	−0.061
1,685	485	1,200	0.379	−0.271	0.389	0.038	−3.699	0.033
1985	485	1,500	0.382	−0.227	0.374	−0.132	−4.211	−0.101
2,285	485	1800	0.263	−0.303	0.297	−0.149	−4.21	−0.192
2,585	485	2,100	0.424	−0.253	0.393	−0.03	−4.451	−0.032

As shown in Table 11, when different numbers of Chinese Holstein cows were added to the joint reference population, the reliability of the total population genomic breeding values for MPY, of Xinjiang Brown cows was 0.28–0.465, with a regression coefficient of 0.277–0.504. The reliability of the genomic breeding values for the validation population was −0.259–0.203, with a regression coefficient of −0.213–0.032.

**TABLE 11:** Genetic parameter estimation of MPY in joint reference population based on ssGBLUP.

Joint reference population	Xinjiang Brown	Chinese holstein	Total population			Validation population		
			R^2^_GEBV_	b_0_	b_1_	R^2^_GEBV_	b_0_	b_1_
485	485	0	0.280	−2.234	0.277	0.203	−1.654	0.136
785	485	300	0.465	−0.307	0.468	−0.066	−4.338	−0.048
1,085	485	600	0.444	−0.185	0.458	−0.158	−5.416	−0.116
1,385	485	900	0.421	−0.17	0.418	0.04	−5.773	0.032
1,685	485	1,200	0.386	−0.28	0.356	−0.091	−6.293	−0.079
1985	485	1,500	0.399	−0.243	0.401	−0.259	−6.967	−0.213
2,285	485	1800	0.397	−0.343	0.504	−0.165	−5.99	−0.162
2,585	485	2,100	0.371	−0.234	0.3	−0.222	−6.578	−0.176

As shown in Table 12, when different numbers of Chinese Holstein cows were added to the joint reference population, the reliability of the total population genomic breeding values for SCS, of Xinjiang Brown cows was 0.190–0.448, with a regression coefficient of 0.207–0.502. The reliability of the genomic breeding values for the validation population was −0.145–0.062, with a regression coefficient of −0.223–0.075.

**TABLE 12:** Genetic parameter estimation of SCS in joint reference population based on ssGBLUP.

Joint reference population	Xinjiang Brown	Chinese holstein	Total population			Validation population		
			R^2^_GEBV_	b_0_	b_1_	R^2^_GEBV_	b_0_	b_1_
485	485	0	0.245	0.011	0.249	0.04	0.007	0.025
785	485	300	0.255	0.01	0.295	−0.145	−0.066	−0.223
1,085	485	600	0.234	0.008	0.232	0.002	−0.071	0.002
1,385	485	900	0.221	0.007	0.207	0.062	−0.084	0.075
1,685	485	1,200	0.448	0.002	0.502	−0.4	−0.084	−0.501
1985	485	1,500	0.248	0.006	0.255	−0.117	−0.089	−0.178
2,285	485	1800	0.254	0.002	0.29	0.005	−0.073	0.006
2,585	485	2,100	0.190	0.003	0.239	−0.093	−0.089	−0.138

## 4 Discussion

### 4.1 Genetic structure analysis

LD is a metric that quantifies whether genotype variations in two SNP markers are relatively consistent and whether they are correlated (Park, 2012). If two loci with adjacent alleles are correlated, certain genotypes tend to be co-inherited, resulting in a higher frequency of certain haplotypes than expected. This pattern can be visually represented using an LD decay plot (Amaral et al., 2008). Because of the correlated inheritance of the two loci, the decay rate of the LD coefficient decreases with increasing generations and recombination events. Genetic background also influences it. Domestication selection reduces genetic diversity within a population, reinforcing the correlation or linkage between SNP loci (Farnir et al., 2000). Consequently, populations with higher degrees of domestication exhibit stronger selection intensities (Odani et al., 2006), resulting in slower LD decay rates. The higher selection intensity in breeding bulls likely reduced the effective population size, thereby affecting LD in these groups. Meanwhile, the LD decay patterns in Xinjiang Brown and Chinese Holstein cows exhibit potential similarities, favoring the construction of a combined reference population. Overall, the LD decayed fastest in Xinjiang Brown cows, indicating lower levels of selection than the other three groups. This suggests a high level of genetic diversity in Xinjiang Brown cows, which harbor rich genetic resources with potential for development and use. These findings provide a scientific basis for the conservation, exploitation, and use of genetic diversity in Xinjiang Brown cattle.

Genetic structure analysis can elucidate phylogenetic relationships and genetic distances among different populations (Whelan and Goldman, 2001). Under the influence of natural and artificial selection, populations exhibiting pronounced genetic differences are evident. When K = 2, there is a distinct genetic structure differentiation among Xinjiang Brown cows, Xinjiang Brown bulls, Brown Swiss bulls, and Chinese Holstein cows. This is related to the breeding strategies employed during the intense selection process of Xinjiang Brown and Chinese Holstein cattle, where most female progenitors in the early stages of population breeding originated from local Chinese yellow cattle (Liu, 2013).

The breeding of Xinjiang Brown and Chinese Holstein cattle involves the introduction of foreign breeds for crossbreeding to improve and enhance the local yellow cattle population in China. Subsequently, through crossbreeding fixation and selective breeding, these breeds have been further developed and stabilized. The genetic background of Xinjiang Brown cattle is traceable to the original crossbreeding improvement in 1951, when the maternal breed was Kazakh cattle (Ma, 2015). Conversely, the genetic background of the Chinese Holstein cattle dates back to 1840. From 1840 to 1948, the Chinese Holstein cattle underwent more than a century of introduction and early stages of crossbreeding improvement. During this period, China initially introduced various dairy breeds, including the Holstein cattle, Jersey cattle, Ayrshire cattle, Brown Swiss cattle, and Shorthorn cattle (Liu, 2013). The distinct genetic structure observed among Xinjiang Brown cows, Xinjiang Brown bulls, Brown Swiss bulls, and Chinese Holstein cows indicates a significant genetic distance between these two major groups. This significantly affects the genetic structure of multi-breed joint reference genomes established later, subsequently affecting the accuracy of genomic predictions.

Figure 5 shows that the coefficient of kinship among individuals within the populations of Xinjiang Brown cows, Xinjiang Brown bulls, and Brown Swiss bulls was relatively high. However, the coefficient of kinship between these groups and the Chinese Holstein cow population tended to be close to 0. This result suggests that when conducting multi-breed genomic selection, it is not advisable to use only one breed as the reference population and the other as the selection population. Instead, it is necessary to fully consider the relationship between the selection and reference populations. When the kinship between the two breeds involved in multi-breed genomic selection is weak or extremely weak, it is necessary to include a certain number of individuals from the same breed in the reference population to ensure high reliability in the estimation of genomic breeding values.

The PCA results identified two major clusters: one containing Xinjiang Brown cows, Xinjiang Brown bulls, Brown Swiss bulls, and Chinese Holstein cows, whereas the other mainly contained Chinese Holstein cows. This suggests a relatively distant genetic relationship between these two clusters. Clustering of Xinjiang Brown cows, Xinjiang Brown bulls, and Brown Swiss bulls is highly concentrated, indicating a close genetic distance among these groups. When considering only the clustering of Chinese Holstein cows, a small subset can be distinguished from the larger population, which exhibits a distant genetic relationship with Xinjiang Brown cows, Xinjiang Brown bulls, and Brown Swiss bulls and with most Chinese Holstein cows. In the subsequent multi-breed genetic evaluations, it is recommended to exclude chip and phenotypic data from this subset of Chinese Holstein cows and their respective farms. This approach can help reduce the interference of genetic structure differences while estimating SNP effects. These findings suggest a low genetic linkage between Xinjiang Brown and Chinese Holstein cattle.

### 4.2 Genetic parameter analysis of the joint reference population

The number of genotyped Chinese Holstein cattle in the joint reference population significantly affects the estimation of variance components. Therefore, it is necessary to consider the number of genotyped animals from different populations in the joint reference population during multi-breed genetic evaluations. The most important factors that affect the reliability of genomic breeding value estimation are the proportion of genetic variance explained by SNPs and trait heritability (Steyn et al., 2019; van Grevenhof et al., 2019). These genetic parameters are directly related to the size and structure of the training population as well as the range, quality, and quantity of phenotypic and genomic information available for individuals in the training population. To ensure accurate genomic breeding value estimation, it is important to minimize the relationship between genotyped individuals within the training population and maximize the relationship between the training and prediction populations. Because ssGBLUP generates genomic breeding values for cows, it is particularly useful for cows with only parental average information. The single-step genomic evaluation combines information from all countries, considering potential duplicate counting of the same information, thereby ensuring more accurate estimation of genomic breeding values. Adding carefully selected cows to the training population can expand the population, improve its structure and relationship with the prediction population, and reduce selection bias. However, in this study, a large proportion of the chip data came from cows, with relatively fewer bulls for validation, probably being a reason for the poor prediction performance.

The ssGBLUP method can simultaneously analyze phenotypic, genomic, and pedigree information from both genotype and non-genotype animals by integrating external information. This method is particularly convenient when using foreign paternal genetic material. For example, during the Interbull evaluation of Brown Swiss bulls, a country can obtain genomic information from multiple countries and MACE information. Combining external MACE information with ssGBLUP can complement paternal information from different countries and provide pseudophenotypic information for foreign paternal lines with no or few offsprings. This research result suggests that we can conduct cross-country genetic evaluations with the Brown Swiss bull origin introduced during the breeding of Xinjiang Brown cattle, which could improve the reliability of genomic predictions for Xinjiang Brown cattle.

### 4.3 Reliability of genetic evaluation in joint reference populations

In theory, a model that assumes the closest distribution of SNP effects to their true distribution can achieve the highest reliability in genomic prediction. The GBLUP model assumes that all SNP effects follow the same normal distribution and compresses the effects of all SNPs to the same degree because different models have different assumptions about the distribution of SNP effects (Villar-hernÁndez et al., 2021). Several methods have been proposed to improve the accuracy of genomic prediction in small populations of dairy cattle (Marjanovic et al., 2021), and one effective approach is to use joint reference populations by combining reference data from different populations (Steyn et al., 2019; van Grevenhof et al., 2019). This method has reported significant benefits in genomic prediction for North American Holstein, European Holstein, Chinese Holstein, and Brown Swiss populations (Vanderick et al., 2017; van den berg et al., 2016). However, the accuracy of genomic prediction is dependent on the relationship between candidate and reference animals, requiring the reference population to be sufficiently close to the target population (Xu et al., 2019). Therefore, for multi-breed joint genetic evaluation between Xinjiang Brown and Chinese Holstein cattle, considering only the added number of animals is insufficient. Further in-depth analysis of important influencing factors, including assumptions about SNP effects (van den berg et al., 2019) and the weights of the A- and G-matrices in the H-matrix (Karaman et al., 2018; Botelho et al., 2021), is required to improve the accuracy and unbiasedness of predictions (Botelho et al., 2021).

Including cows in the genotyping reference population is necessary because of the limited number of cows with reliable phenotypic information available for predicting offspring traits. To improve the genomic breeding values of the population, it is necessary to include a certain number of validated bulls with reliable phenotypic information in the reference population (Vanraden et al., 2020). Previous studies have reported that the inclusion of cows in the validated bull reference population can improve the accuracy of genomic prediction (Ding et al., 2013). Although the phenotypic information for cows is less accurate than that for bulls with offspring validation, additional information can still be significant (Cole et al., 2021), considering the large number of cows available as reference animals.

## 5 Conclusion

The genetic structure of mature Xinjiang Brown and Chinese Holstein cows is different, and the individual kinship between these two populations is relatively distant. This increases the impact of genetic structure and kinship on the reliability of genomic breeding value estimation. Through comparisons of parameters, including heritability, breeding value reliability, and unbiasedness, it was initially determined that including 600 and 900 Chinese Holstein cows in the joint reference population positively impacted the genomic prediction of Xinjiang Brown cattle to some extent. In multi-breed genome selection, it is necessary to pre-evaluate the genetic structure and genetic relationship of the population. It is feasible to combine the Chinese Holstein cattle population into the Xinjiang brown cattle population to form a joint reference group for cross-breed genetic assessment. It can provide theoretical guidance for applied genomic genetic assessment and multi-breed genomic genetic assessment of Xinjiang brown cattle, and also provide reference for genome selection of other dual-use cattle and small population breeds.

## Data Availability

The raw data supporting the conclusion of this article will be made available by the authors, without undue reservation.
